# Characterization of different fat depots in NAFLD using inflammation-associated proteome, lipidome and metabolome

**DOI:** 10.1038/s41598-018-31865-w

**Published:** 2018-09-21

**Authors:** Alen Lovric, Marit Granér, Elias Bjornson, Muhammad Arif, Rui Benfeitas, Kristofer Nyman, Marcus Ståhlman, Markku O. Pentikäinen, Jesper Lundbom, Antti Hakkarainen, Reijo Sirén, Markku S. Nieminen, Nina Lundbom, Kirsi Lauerma, Marja-Riitta Taskinen, Adil Mardinoglu, Jan Boren

**Affiliations:** 1 Science for Life Laboratory, KTH - Royal Institute of Technology, Stockholm, Sweden; 2 Heart and Lung Center, Division of Cardiology, Helsinki University Central Hospital and University of Helsinki, Helsinki, Finland; 3 Department of Biology and Biological Engineering, Chalmers University of Technology, Gothenburg, Sweden; 4 Department of Molecular and Clinical Medicine/Wallenberg Lab, University of Gothenburg, and Sahlgrenska University Hospital, Gothenburg, Sweden; 5 Department of Radiology, HUS Medical Imaging Center, Helsinki University Central Hospital and University of Helsinki, Helsinki, Finland; 6 Department of General Practice and Primary Health Care, Health Care Centre of City of Helsinki and University of Helsinki, Helsinki, Finland

## Abstract

Non-alcoholic fatty liver disease (NAFLD) is recognized as a liver manifestation of metabolic syndrome, accompanied with excessive fat accumulation in the liver and other vital organs. Ectopic fat accumulation was previously associated with negative effects at the systemic and local level in the human body. Thus, we aimed to identify and assess the predictive capability of novel potential metabolic biomarkers for ectopic fat depots in non-diabetic men with NAFLD, using the inflammation-associated proteome, lipidome and metabolome. Myocardial and hepatic triglycerides were measured with magnetic spectroscopy while function of left ventricle, pericardial and epicardial fat, subcutaneous and visceral adipose tissue were measured with magnetic resonance imaging. Measured ectopic fat depots were profiled and predicted using a Random Forest algorithm, and by estimating the Area Under the Receiver Operating Characteristic curves. We have identified distinct metabolic signatures of fat depots in the liver (TAG50:1, glutamate, diSM18:0 and CE20:3), pericardium (N-palmitoyl-sphinganine, HGF, diSM18:0, glutamate, and TNFSF14), epicardium (sphingomyelin, CE20:3, PC38:3 and TNFSF14), and myocardium (CE20:3, LAPTGF-β1, glutamate and glucose). Our analyses highlighted non-invasive biomarkers that accurately predict ectopic fat depots, and reflect their distinct metabolic signatures in subjects with NAFLD.

## Introduction

NAFLD is recognized as the most prevalent liver disorder in Western countries, predicted to become the major cause for liver transplantation in the near future^1^. NAFLD encompasses the spectrum of chronic liver disorders ranging from asymptomatic non-alcoholic steatosis, defined by liver enlargement and abnormal accumulation of lipids without inflammation, to NASH, a more clinically significant form of NAFLD. The latter is characterized by hepatic inflammation and fibrosis that can eventually lead to cirrhosis and hepatocellular carcinoma^1,2^. NAFLD is also linked to metabolic syndrome (MetS), a cluster of risk factors including central obesity, high blood pressure, elevated level of fasting plasma glucose, high serum triglycerides (TGs) and low levels of high-density lipoproteins (HDL). These complications are also well known causative factors of insulin resistance (IR), type 2 diabetes (T2D) and cardiovascular disease (CVD)^3–5^. The association between NAFLD and CVD is of particular interest as numerous studies pointed out to an increased prevalence of heart related conditions among NAFLD patients^6,7^. Specifically, under liver steatosis, the cardiovascular system is compromised, exhibiting structural alterations and remodeling^8^. For example, animal studies have shown that intracellular lipid accumulation in the myocardium and other toxic products of fatty acid (FA) metabolism causes cardiac lipotoxicity, impairs left ventricular (LV) function, and promotes cardiac fibrosis and apoptosis^9^. Myocardial TG content is higher in subjects with obesity^10^, MetS^11^, impaired glucose tolerance^12^ or T2D^13,14^, suggesting that cardiac steatosis is relatively common in humans. Importantly, these increases in intramyocardial TG levels precede the development of cardiac dysfunction, suggesting a causative role of myocardial fat in the development of obesity/glucose intolerance/T2D-induced cardiac dysfunction^14,15^.

It has been reported that cardiac fat accumulation contributes to the high mortality following myocardial infarction in subjects with T2D^16^. However, despite an increasing body of evidence showing an association between elevated myocardial TG content and cardiac dysfunction in humans^17^, it remains largely unclear whether and how alterations in myocardial TG synthesis and hydrolysis affect heart function. In fact, we have previously shown that in obese, non-diabetic men only accumulation of epicardial and pericardial, but not of myocardial TG, was linked to the severity of structural and functional alterations of the heart^18^. Although myocardial TG content is recognized as a site of ectopic fat accumulation, it may be regulated by other factors than those linked to systemic fat depositions.

Over the past decade new technologies that embrace the term –omics have evolved to address increasingly complex biological questions arising out of the postgenomic era. Analysis of small metabolites and lipids is expected to provide many more additional insights and clues to the mechanisms of biological processes and functions, and thus may increase our knowledge of the development and progression of CVD. Here, we determined the independent role of a broad panel of parameters comprising plasma proteins associated with inflammation as well as plasma lipid (lipidome) and metabolite (metabolome) signatures in the occurrence of myocardial, epicardial, pericardial and liver fat among male subjects with NAFLD.

## Results

### Association of clinical parameters with various fat depots

Ectopic fat (myocardial, epicardial, pericardial and liver) was measured in non-diabetic male subjects with NAFLD, which were split into high and low fat groups. Clinical and biochemical features of the subjects are summarized in Supplementary Table 1. Subjects with high level of ectopic fat tended to present characteristics of MetS (myocardial: 72% subjects; epicardial: 76%; pericardial: 88%: liver: 92%), and were overall older with increased BMI, waist circumference, IR and more pronounced smoking habits compared to subjects with low level of ectopic fat. Serum level of TG, total cholesterol, low density lipoprotein (LDL), very low density lipoprotein, apolipoprotein B (apoB) were also significantly increased in subjects with high level of ectopic fat. In turn, the levels of HDL tend to be lower with increasing levels of measured ectopic fat. HOMA-index, plasma glucose and insulin levels, as well as their levels after first and second hour during OGTT, were higher among subjects with high level of ectopic fat.

Serum concentrations of potential biomarkers for cardiac fat depots (beta-hydroxybutyrate (β-OHB), fatty acid binding protein (AFABP), leptin and adiponectin) previously proposed by (Graner *et al*. 2015) demonstrated similar behaviour across the measured ectopic fat depots. For instance, AFABP and leptin were significantly higher among subjects with high ectopic fat, whereas adiponectin levels were significantly lower, compared to subjects with low ectopic fat depots. β-OHB levels were found to be significantly lower among individuals with high levels of myocardial and epicardial fat (Supplementary Table 1).

**Figure 1:**
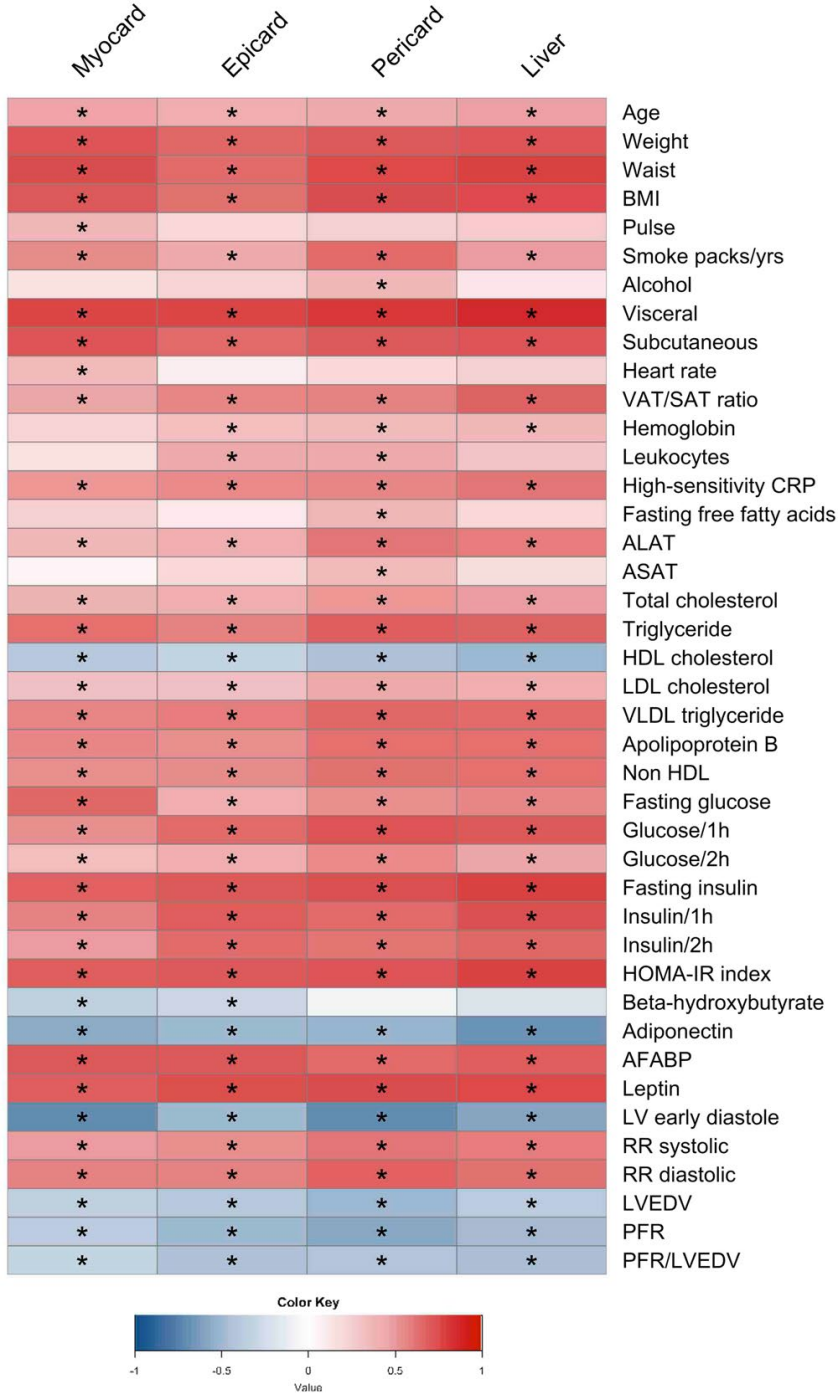
Heatmap visualization based on Spearman correlation between variables of interest (column wise) and clinical parameters (row wise). Colour key indicates strength of a relationship: blue colour – negative relationship, red colour – positive relationship. *Significant relationship after FDR correction and significance level of 0.05.

We have performed an association analysis (Fig. 1) and observed that visceral and subcutaneous fat, waist circumference, BMI, AFABP, HOMA-index, leptin, fasting glucose and insulin, TG levels, systolic and diastolic blood pressures were positively correlated with the levels of measured fat depots whereas adiponectin, HDL and β-OHB showed negative correlations. Furthermore, negative correlations were observed between LV early diastole, peak filing rate (PFR), LV end-diastolic volume (LVEDV), PFR/LVEDV and high levels of measured ectopic fat. Our association analysis also showed that among ectopic fat depots around the heart, pericardial fat showed the strongest correlation with the clinical status of subjects involved in this study, significantly correlating with majority of the clinical parameters analysed.

### Relationship of inflammation-associated proteome and various fat depots

**Figure 2:**
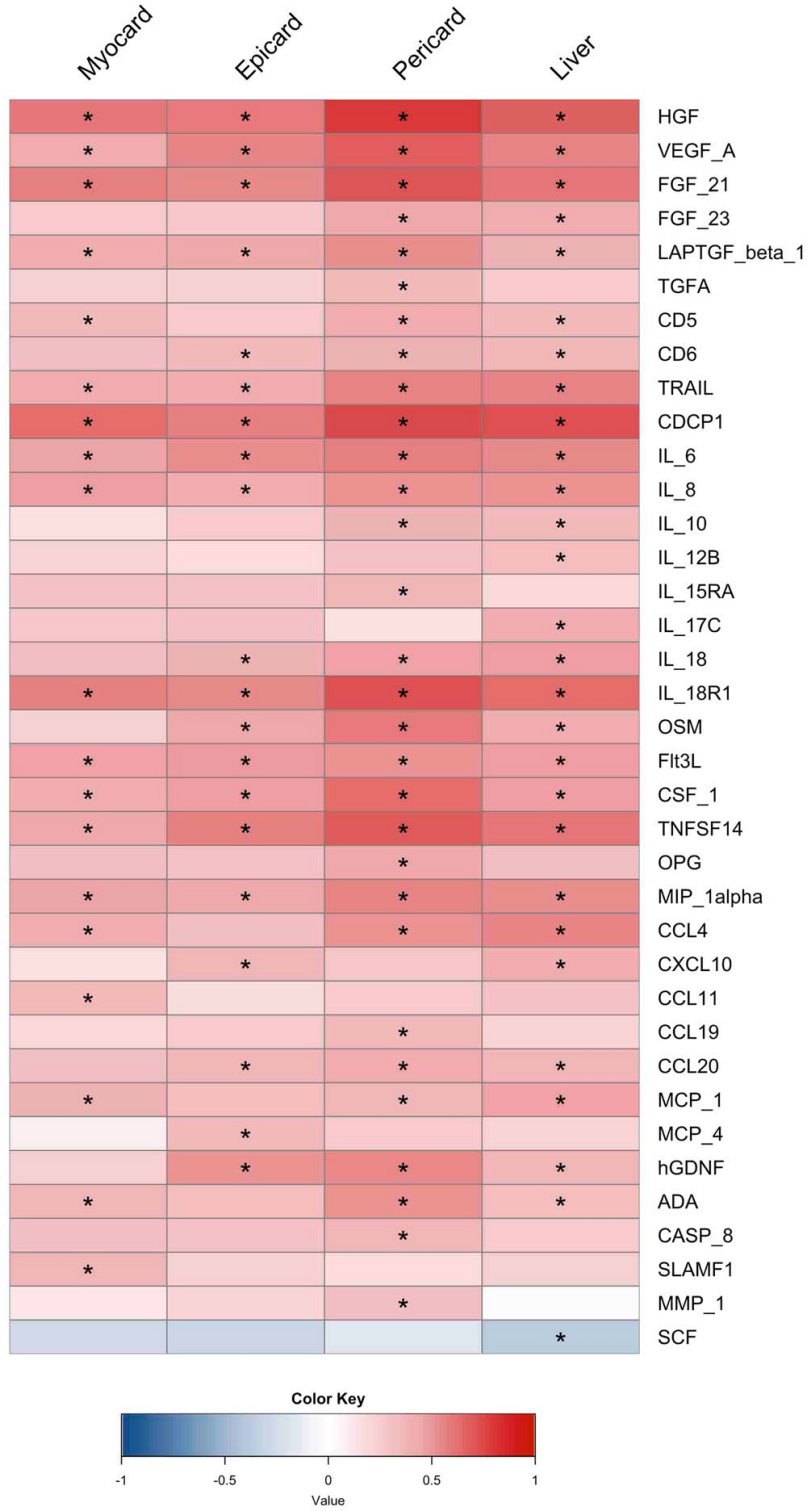
Heatmap visualization based on Spearman correlation between variables of interest (column wise) and inflammation-associated proteome (row wise). Colour key indicates strength of a relationship: blue colour – negative relationship, red colour – positive relationship. *Significant relationship after FDR correction and significance level of 0.05.

In order to understand the link between measured ectopic fat and inflammation, we quantified a range of biomarkers associated with inflammation. We used a multiplex immunoassay enabling analysis of 92 inflammation-related protein biomarkers. The relationship between these biomarkers and measured ectopic fat depots are presented in Fig. 2. We observed that pericardial fat displayed the highest correlation with inflammation-related proteins whose levels significantly differed between subjects with high and low ectopic fat.

Based on our analysis, pericardial fat showed significant positive correlation with several growth factor proteins including hepatocyte growth factor (HGF), vascular endothelial growth factor A (VEGF-A), fibroblast growth factors 21 and 23 (FGF21 and FGF23), transforming growth factor β1 (LAPTGF-β1) and transforming growth factor A (TGFA). We have also observed that other fat depots including myocardial, epicardial and liver fat exhibited similar trend with these growth factor proteins. However, these associations were weaker than those of pericardial fat. In addition, we found that there is no significant correlation between TGFA and myocardial, epicardial and liver fat or between FGF23 and myocardial and epicardial fat.

We have also observed high correlations between pericardial fat and the levels of various differentiation clusters (CD5, CD6, tumour necrosis factor-related apoptosis-inducing ligand TRAIL, i.e. CD253, and CUB domain containing protein 1 CDCP1, i.e. CD318). On the other hand, we found no significant association between increasing levels of CD5 and epicardial fat, as well as CD6 and myocardial fat. Among different interleukin families, only IL6, IL8 and IL18R1 were significantly correlated with each fat depot while the highest correlation was again observed with pericardial fat. Increasing levels of epicardial, pericardial and liver fat showed significant correlations with IL18, whereas levels of IL10 had significant correlations with pericardial and liver fat.

Furthermore, eight different inflammatory chemokines showed a significant positive correlation with at least one of the measured ectopic fat depots. Closely-related chemokines such as MIP-1α (CCL3) and MIP-1β (CCL4) presented identical trends and very similar relationships with measured ectopic fat depots. MCP1 (CCL2) presented significant positive association with all measured ectopic fat depots except epicardial fat. CXCL10 was found to have significant relationship only with liver and epicardial fat. Chemokines including CCL11, MCP4 (CCL13) and CCL19 showed significant positive correlations with at least one of the measured ectopic fat depots.

Interestingly, we found that serum levels of other inflammatory proteins including oncostatin (OSM), FMS-like tyrosine kinase 3 ligand (FLT3L), tumour necrosis factor superfamily member 14 (TNFSF14), osteoprotegerin (OPG), glial cell-derived neurotrophic factor (hGDNF), adenosine deaminase (ADA), caspase-8 (CASP8) and matrix metalloproteinase-1 (MMP-1) presented the strongest positive association with pericardial fat. Multi-protein E3 ubiquitin ligase complex (SCF) was the only inflammation factor showing negative correlation with any fat depot (liver fat).

Our analysis indicated that a high ectopic fat level correlates intimately with pro-inflammatory factors/mediators as well as cancer related plasma proteins. In order to investigate the tissue of origin for each secreted protein, we checked the mRNA expression level of each protein in major human tissues using the data presented in Human Protein Atlas^19–21^, specifically expressed (tissue enriched, tissue enhanced, group enriched) either in liver, adipose tissue or heart muscle. Among all genes, 3 are specific to these tissues: FGF21, IL6, and CCL19. FGF21 exhibited a high correlation with liver fat and is a liver tissue enriched gene. In turn, IL6 is significantly correlated with pericardial fat and is enhanced to adipose tissue. Finally, CCL19 is significantly correlated with pericardial fat and displays enriched expression in adipose tissue among other tissues. In addition, though TNFSF14 and CSF1 are found in multiple tissues, their highest expression is observed in adipose tissue (Supplementary Table 2).

### Association of lipidome and various fat depots

**Figure 3:**
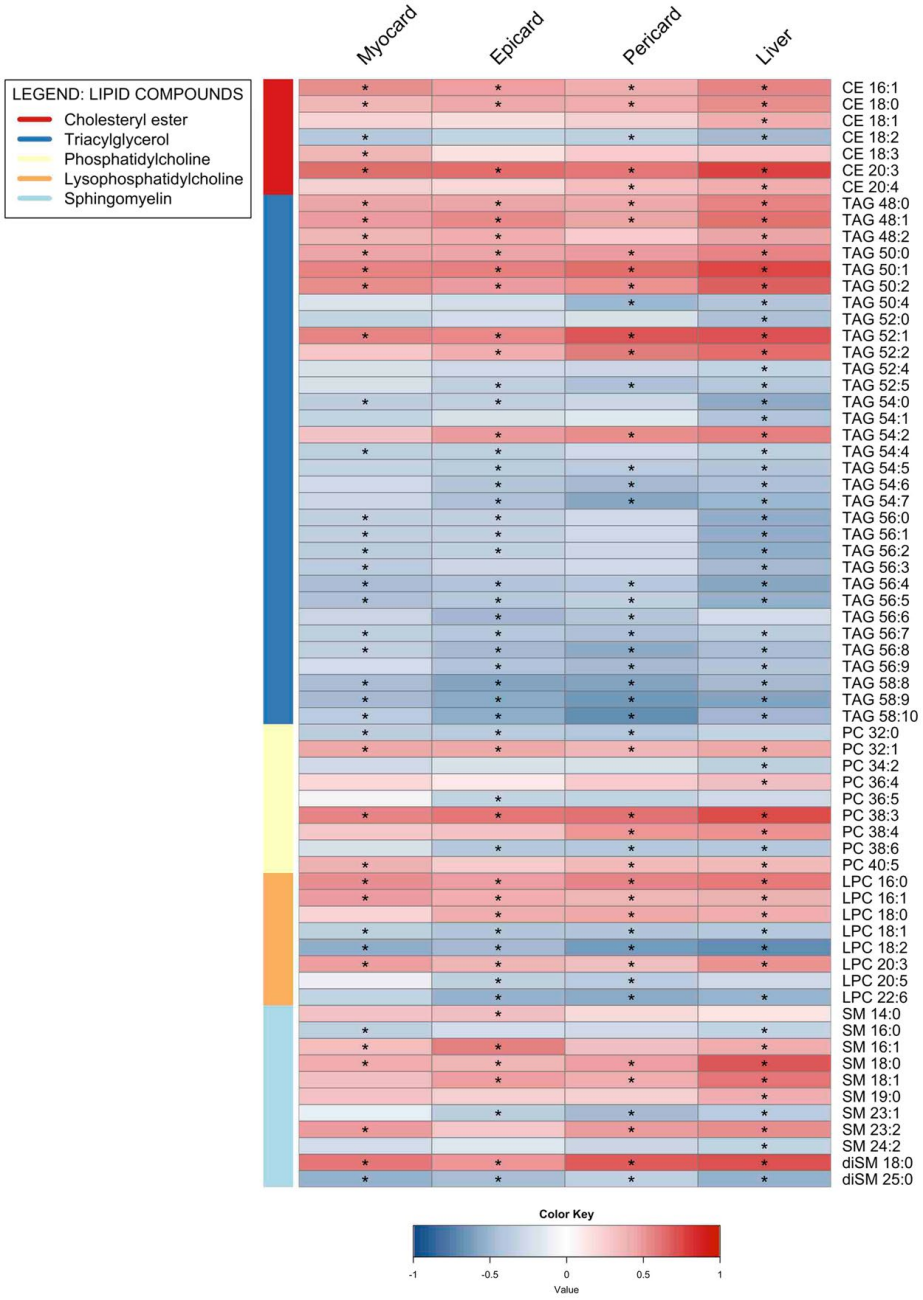
Heatmap visualization based on Spearman correlation between variables of interest (column wise) and lipidome (row wise). Colour key indicates strength of a relationship: blue color – negative relationship, red colour – positive relationship. *Significant relationship after FDR correction and significance level of 0.05.

We then sought to identify significant associations between lipid classes and ectopic fat depots and observed significant correlations involving five different lipid classes including cholesterol ester (CE), triacylglycerol (TAG), phosphatidylcholine (PC), lysophosphatidylcholine (LPC) and sphingomyelin (SM). Altogether, 67 different lipid species were found to be associated with the measured ectopic fat depots (Fig. 3). TAGs were the most abundant class in terms of number, with 32 unique species significantly correlating with at least one observed ectopic fat. TAGs containing fatty acids (FA) with C48 to C50 total carbon atoms regardless of saturation showed strong positive correlations with measured ectopic fat depots. The exception were TAG (50:4) species which showed negative correlations with ectopic fat depots (liver and pericard). TAGs possessing FA with C52 to C58 showed overall negative correlations, with exception of TAGs (52:1, 52:2, 54:2) that showed positive correlations with some of the ectopic fat depots. SM was the second most abundant class detected in this analysis, and almost 3/4 of species showed positive correlations with ectopic fat depots. From the SM pool, diSM (18:0) stands out with the highest positive correlation towards ectopic fat depots. Members of different PC species showed both positive and negative associations with measured fat depots. PC (C36: and C38:) species presented a shift in correlations with fat depots from positive to negative as the number of double bounds increased while, PC (C32:) species showed the opposite behaviour. All CE species but CE (18:2) were found to be positively correlated with increasing levels of fat in measured ectopic fat depots. Furthermore, CEs (20:3) consistently showed strong correlations with the measured ectopic fat depots. Our analysis showed that liver fat had the highest absolute correlation with the changes in the lipidome (Fig. 3).

### Metabolome characteristics

**Figure 4:**
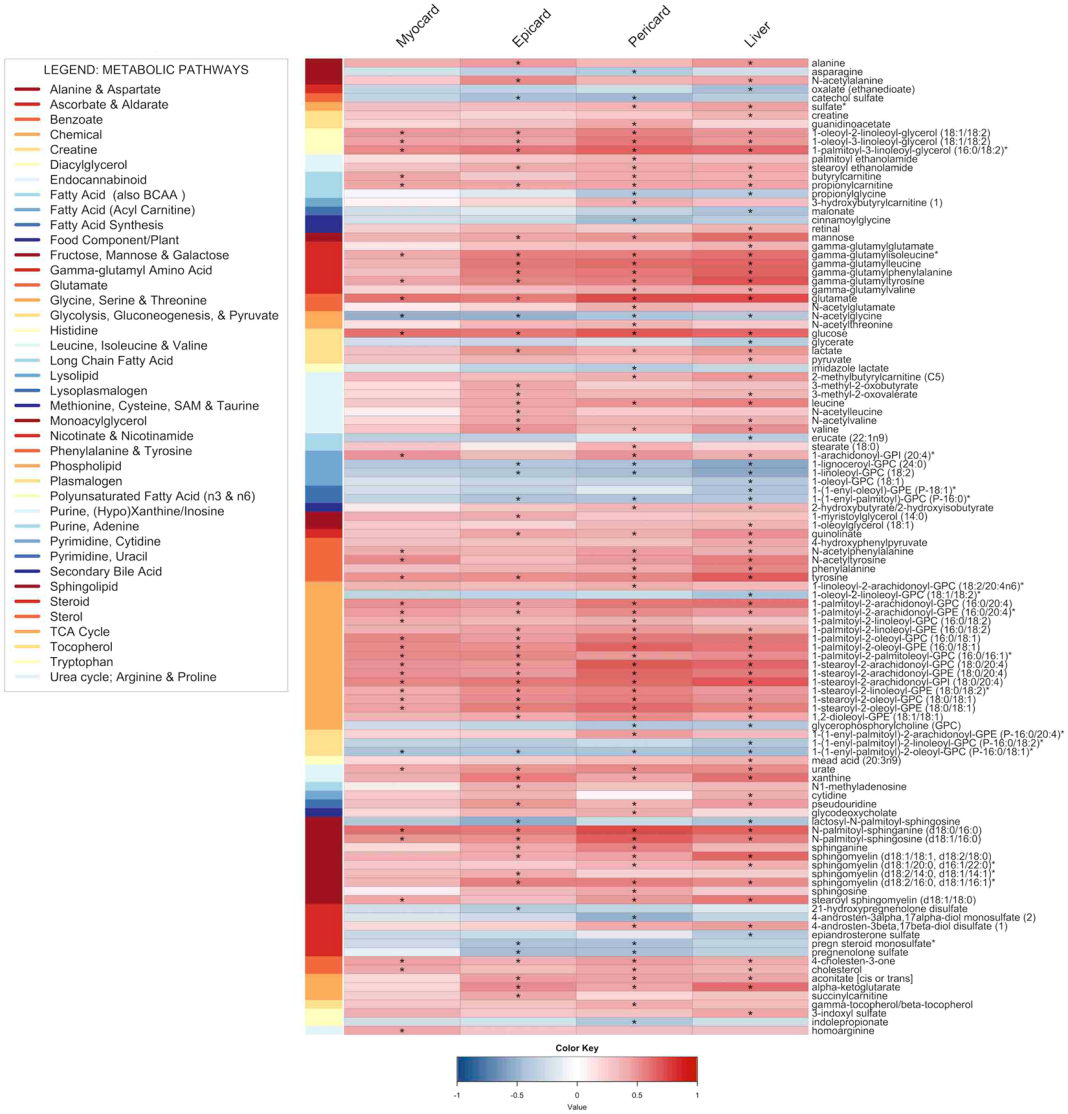
Heatmap visualization based on Spearman correlation between variables of interest (column wise) and metabolome (row wise). Colour key indicates strength of a relationship: blue colour – negative relationship, red colour – positive relationship. *Significant relationship after FDR correction and significance level of 0.05.

To understand the individual role of each metabolite, we performed an untargeted metabolomics study using the platform by Metabolon Inc., as previously described^22^. Metabolome screening revealed eight metabolic pathways (amino acid, carbohydrate, cofactor/vitamin, energy, lipid, nucleotide, peptide and xenobiotics) significantly associated with ectopic fat depots. Major pathways were further classified into 40 different metabolic sub-pathways, exhibiting the magnitude of altered metabolome as a consequence of obesity (Fig. 4**)**. Here, correlation analysis revealed both positive and negative association between compounds involved in amino acid metabolism and measured ectopic fat. The level of metabolites related to carbohydrate metabolism specifically in pathways responsible for metabolism of carbohydrates like glucose, mannose, lactate, pyruvate, aconitate (cis or trans), α-ketoglutarate and succinylcarnitine showed positive correlations with accumulation of ectopic fat depots. Compounds related to lipid metabolism were found to be altered markedly in terms of numbers (60) and extent of different lipid sub-pathways (16), including phospholipid metabolism and sphingolipid metabolism. Glycerophosphoryl cholines (GPC), ethilonamines (GPE) and inositols (GPI) were highly correlated with the level of fat in the measured depots. On the other hand, the plasma level of GPC (18:1/18:2) and α-GPC were negatively associated with the measured ectopic fat depots.

Besides major changes in some phospholipid sub-pathways, alterations were observed in monoacylglycerol, diacylglycerol, endocannabinoid, polyunsaturated fatty acid (n3 and n6), secondary bile acid, and sterol metabolites that showed overall positive correlations with the measured ectopic fat depots. FAs and related metabolites showed both positive and negative correlations with fat depots, whereas lysolipid, lysoplasmalogen, plasmalogen and steroid metabolism showed overall negative associations with minor exceptions (Fig. 4**)**. In addition, metabolites in both nucleotide and peptide metabolism showed overall positive correlations with the level of measured ectopic fat. Overall, the metabolites with the strongest positive correlations across the measured ectopic fat depots were glutamate, N-palmitoyl-sphinganine (CER (d18:0/16:0)), glucose, 1-palmitoyl-3-linoleoyl-glycerol (16:0/18:2) and 1-stearoyl-2-arachidonoyl-GPI (18:0/20:4).

### Identifying the key variables for predicting fat depots

**Figure 5:**
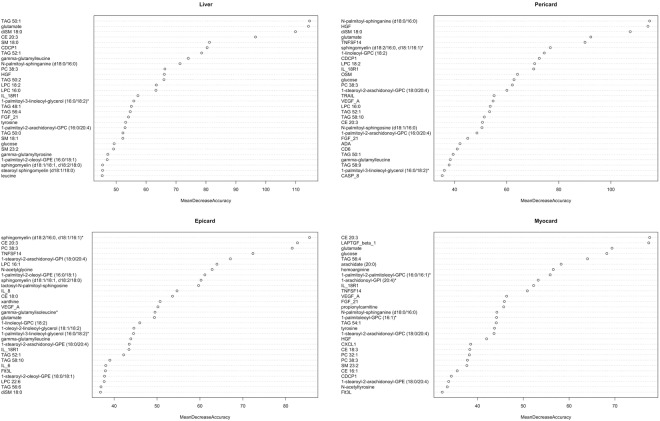
RF analysis (combined dataset) – variable importance based on mean decrease in accuracy for liver, pericardial, epicardial and myocardial fat.

Finally, we sought to identify biomarkers with high prediction capabilities for different ectopic fat depots in patients with diagnosed NAFLD using machine learning. We used a Random Forest (RF) algorithm for feature selection based on mean decrease of accuracy (MDA). This analysis was performed for each variable of interest (myocardial, epicardial, pericardial and liver fat) across four different datasets (clinical, inflammation-associated proteome, lipidome and metabolome). Top 15 variables predicted by the RF (Table 1 – top five, details in Supplementary Table 3), we then evaluated for their individual classification ability by estimating AUC (Table 2 – highest AUC score, details in Supplementary Table 4). The top five covariates from the ROC analysis across the datasets showed high classification ability (AUC score 0.80 to 0.98). Of the different heart fat depots, pericardial fat was predicted with the highest classification ability across datasets (waist, AUC = 0.98; HGF, AUC = 0.96; diSM 18:0, AUC = 0.94; and CER (d18:0/16:0), AUC = 0.94). Considering all the measured ectopic fat depots, liver fat was predicted with the highest classification ability across dataset (HOMA-index, AUC = 0.98; diSM 18:0, AUC = 0.96; and glutamate, AUC = 0.95), with exception of inflammation-associated proteome (CDCP1, AUC = 0.93). Rather than to compute whether differences in AUC between individual covariates are statistically significant, we have combined all covariates (Supplementary Table 4, excluding clinical covariates) in a single dataset and evaluated their classification importance with RF based on MDA (Fig. 5). As the permutation of important covariate greatly affects overall accuracy of the RF model, covariates more important for classification will have higher MDA. RF analysis with the combined dataset identified several covariate combinations as the most important for classification. Specifically, analysis identified four covariates (TAG 50:1, glutamate, diSM 18:0, CE 20:3) in the liver fat classification, five covariates (CER (d18.0/16.0), HGF, diSM 18:0, glutamate, TNFSF14) in pericardial fat classification, four covariates (SM (d18:2/16:0, d18:1/16:1), CE 20:3, PC 38:3, TNFSF14) in epicardial fat classification, and four covariates (CE 20:3, LAPTGF-β1, glutamate, glucose) in myocardial fat classification (Fig. 5).

**Table 1:** RF results of top 5 most important biomarkers (mean decrease in accuracy).

Myocardial TG	Epicardial fat	Pericardial fat	Liver fat
**Clinical Data**			
LV early diastole	Visceral fat	Waist	Waist
Waist	Leptin	Visceral fat	Fasting insulin
Weight	Insulin 1 h	Leptin	HOMA-index
AFABP	AFABP	Fasting insulin	Visceral fat
BMI	Fasting insulin	HOMA-index	Weight
**Inflammation-Associated Proteome**			
FGF 21	TNFSF 14	HGF	CDCP1
LAPTGF-β1	FGF 21	CDCP1	FGF 21
IL 18R1	IL 18R1	TNFSF 14	HGF
HGF	HGF	TRAIL	IL 18R1
CDCP1	IL 6	IL 18R1	IL 6
**Lipidome**			
CE 20:3	CE 20:3	diSM 18:0	TAG 50:1
TAG 56:4	PC 38:3	LPC 18:2	diSM 18:0
PC 38:3	LPC 16:1	PC 38:3	CE 20:3
LPC 18:2	TAG 52:1	LPC 16:0	SM 18:0
CE 16:1	CE 18:0	CE 20:3	TAG 52:1
**Metabolome**			
Glutamate	^*^PE (16:0/18:1)	^*^CER (d18:0/16:0)	Glutamate
Glucose	^*^PI (18:0/20:4)	Glutamate	γ- glutamyl-leucine
^*^DAG (16:0/18:2)	Glutamate	Glucose	^*^CER (d18:0/16:0)
^*^PE (18:0/20:4)	^*^PE (18:0/20:4)	^*^PC (18:0/20:4)	^*^DAG (16:0/18:2)
^*^PC (16:0/16:1)	^*^SM (d18:2/16:0, d18:1/16:1)	^*^SM (d18:2/16:0, d18:1/16:1)	^*^PE (16:0/18:1)

*DAG (16:0/18:2) – 1-palmitoyl-3-linoleoyl-glycerol (16:0/18:2); *PE (18:0/20:4) – 1-stearoyl-2-arachidonoyl-GPE (18:0/20:4); *PC (16:0/16:1) – 1-palmitoyl-2-palmitoleoyl-GPC (16:0/16:1); *PE (16:0/18:1) – 1-palmitoyl-2-oleoyl-GPE (16:0/18:1); *PI (18:0/20:4) – 1-stearoyl-2-arachidonoyl-GPI (18:0/20:4); ‘SM (d18:2/16:0, d18:1/16:1) – sphingomyelin (d18:2/16:0, d18:1/16:1); *CER (d18:0/16:0) – N-palmitoyl-sphinganine (d18:0/16:0); *PC (18:0/20:4) – 1-stearoyl-2-arachidonoyl-GPC (18:0/20:4).

**Table 2:** ROC analysis of potential biomarkers with the highest AUC scores from analysed datasets.

Predictors	AUC	95% confidence interval		SN	SP	Threshold
		Lower limit	Upper limit			
**Myocardial TG**						
Waist	0.905	0.810	1.000	0.92	0.84	90.5 cm
CDCP1	0.837	0.725	0.949	0.80	0.76	3.31
CE 20:3	0.858	0.748	0.967	0.88	0.80	0.804
Glucose	0.845	0.734	0.955	0.76	0.84	3.5 × 10^8^
**Epicardial FAT**						
Visceral fat	0.952	0.899	1.000	0.92	0.88	1373 mm^2^
IL - 18R1	0.853	0.748	0.957	0.84	0.80	6.69
CE 20:3	0.888	0.793	0.983	0.92	0.76	0.832
^*^PE (18:0/20:4)	0.850	0.734	0.965	0.80	0.88	6.5 × 10^5^
**Pericardial FAT**						
Waist	0.975	0.927	1.000	1.00	0.92	93 cm
HGF	0.963	0.919	1.000	0.96	0.88	6.79
diSM 18:0	0.938	0.864	1.000	0.92	0.88	0.225
^*^CER (d18:0/16:0)	0.942	0.869	1.000	1.00	0.8	8.7 × 10^5^
**Liver FAT**						
Visceral fat	0.984	0.960	1.000	0.92	0.96	1588.5 mm^2^
CDCP1	0.926	0.844	1.000	0.84	0.96	3.50
diSM 18:0	0.963	0.917	1.000	0.92	0.88	0.226
Glutamate	0.946	0.874	1.000	0.96	0.92	2.9 × 10^8^

Unless otherwise stated, all threshold values represent relative units. *PE (18:0/20:4) – 1-stearoyl-2-arachidonoyl-GPE (18:0/20:4); *CER (d18:0/16:0) – N-palmitoyl-sphinganine (d18:0/16:0).

## Discussion

In this study, we expanded our previous investigation on biomarkers and prediction of myocardial TG content^8^ by integrating myocardial, epicardial, pericardial and liver ectopic fat depots with inflammation-associated proteome, lipidome and metabolome. Using single and combined multiple omics data in this cohort of non-diabetic men with diagnosed NAFLD, we performed association and classification analyses where we investigated which and how markers in the proteome, lipidome, metabolome, as well as their combination, would associate and predict fat depot accumulation. For instance, our association analysis showed significantly higher levels of well-known risk factors of MetS, T2D and NAFLD, namely AFABP, APOB, HOMA-index, LDL, leptin and TG among subjects with elevated levels of the measured ectopic fat depots. Overall, the association and classification analyses are highly consistent and show the power of individual markers to classify and discriminate ectopic fat depots. Further, in addition to validating known obesity markers, we identified a novel signatures to classify and discriminate ectopic fat depots.

Certain biomarkers performed very well in discriminating all measured ectopic fat depots whereas others discriminated only specific ones. Individually, visceral fat, HGF, IL-18R1, TNFSF14, CE20:3 and glutamate ranked among the top five in discriminating all of the measured ectopic fat depots while waist, fasting insulin and HOMA-index, CDCP1, LPC 18:2, PC38:3, diSM 18:0 and CER (d18:0/16:0) ranked in top five discriminating at least three ectopic fat depots. In addition, we have used a combined dataset of individual biomarkers to identify plasma metabolic profiles comprised of biomarkers that robustly classified ectopic fat depots. Our analysis revealed several lipid species, inflammation related proteins and single amino acid as a metabolic signature of measured ectopic fat depots. For instance, the liver fat depot is marked mainly with different lipid species (TAG50:1, diSM18:0, and CE20:3) and glutamate, while the metabolic signature of pericardial fat depot is mainly presented by elevated levels of CER (d18:0/16:0), diSM18:0, glutamate, HGF and TNFSF14. Furthermore, we showed that the metabolic signature of epicardial fat depot is characterized with augmented levels of SM (d18:2/16:0, d18:1/16:1), CE20:3, PC38:3 and TNFSF14, while the metabolic signature of myocardial fat depot is mainly composed with changes in CE20:3, LAPTGF-β1, glutamate and glucose.

Adiponectin, a highly expressed cytokine by the adipose tissue^20^, with known anti-atherogenic, anti-diabetic and anti-inflammatory properties^23^, showed significantly reduced serum levels among the subjects with elevated levels of measured ectopic fat depots. This is in agreement with previous obesity related studies reporting decreased serum levels of adiponectin among overweighed subjects^24^. Moreover, our study showed strong inverse associations between PFR or LVEDV and ectopic fat depots. Together with the observed positive association of systolic and diastolic blood pressures and ectopic fat depot levels, our data suggests that functional and structural deterioration of the heart follows accumulation of ectopic fat, as reported previously^25,26^.

It is well known that the adipose tissue is not only an energy storage but rather a complex and highly active metabolic and endocrine organ. Thus, serum concentration of inflammation-related proteins can reflect the profiles of measured ectopic fat depots, and serve as potentially good classifiers^27^. Accordingly, our association analysis highlighted a strong relationship between several growth factors, clusters of differentiation and inflammatory cytokines/chemokines and the levels of ectopic fat depots. Among these FGF21, IL-6, CCL19, TNFSF14 and CSF1 were of particular interest due to their high specificity in liver, adipose tissue or cardiac muscle.

FGF21 is expressed in the liver and plays an important role in regulating lipid and glucose metabolism. Several studies have shown that FGF21 overexpression in rodents is associated with weight loss and lower obesity, as well as insulin sensitivity in its target tissues^20,28,29^. However, the metabolic role of FGF21 in humans is not completely clear. Human studies have reported a positive association of FGF21 serum levels with obesity and ectopic fat depots^30–33^, which is in agreement with our observations, as well as great variability in FGF21 serum levels^34,35^. Our classification analysis highlighted FGF21 as a good individual classifier of measured ectopic fat depots, but not as valuable component of their metabolic signatures.

IL6 expression in adipose tissue, its positive association with obesity, systemic IR and inflammation is in agreement with our observations^20,36–38^, but its role in obesity and chronic low-grade inflammation is poorly understood. It had been observed that IL6 exhibits dual effect: during acute inflammation, IL6 plays a protective role by controlling the expression levels of other pro-inflammatory cytokines. However, IL6 becomes pro-inflammatory under chronic inflammation by promoting B-cell differentiation and T-cell activation^39–41^. Here, IL6 performed well as an individual classifier, discriminating all measured ectopic fat depots, but poor in representing their metabolic signatures.

CCL19 is a chemokine expressed in adipose tissues that is believed to play an important role in low-grade inflammation observed in obesity by recruiting macrophages to those tissues^20,42^. Animal models showed associations of the CCL19-CCR7 pathway with obesity-associated inflammation and IR^43^. Despite these observations, CCL19 ranked low for discriminating measured ectopic fat depots.

TNFSF14 is a member of the TNF superfamily, expressed in various human tissues including adipose^20,44^. TNFSF14/HVEM (herpesvirus entry mediator) are involved in obesity-induced inflammation by recruiting and enhancing macrophage lineage in adipose tissue, thus subsequently responsible for the release of several pro-inflammatory cytokines including TNFα, IL6 and MCP-1^45^. Accordingly, significantly changed serum levels of TNFSF14 have been observed among obese subjects^44^ which is in agreement with our observation. Here, our discrimination analysis highlighted TNFSF14 as an excellent individual classifier of pericardial fat, good classifier of epicardial and liver fat, and moderate classifier of myocardial fat. In addition, it was found as a valuable metabolic signature component of pericardial and epicardial fat.

CSF1 is a cytokine expressed in a variety of tissues including adipose^20^. Studies in human and animal models proposed a role of CSF1 in proliferation, differentiation and macrophage survival once recruited into adipose tissue. Most of the recruited macrophages present in the adipose tissue of obese subjects exhibit high expression of inflammatory factors, and the macrophage number shows strong positive association with adiposity^46^. Accordingly, CSF1 expression is upregulated in growing adipose tissue^47^. Though our observations confirmed a positive association with obesity, CSF1 did not perform well in discriminating ectopic fat depots.

Metabolic disturbance including amino acid, carbohydrate and lipid metabolism are linked with central and peripheral obesity^48^. Although the underlying pathways are not well understood, recent studies offered new insights into the aetiology and relationship between metabolic perturbations and obesity^49–51^. Here, we showed an association between several compounds associated with amino acid metabolism and measured ectopic fat depots, among which glutamate presented the strongest association. Moreover, we observed that this is the only compound associated with amino acid metabolism that displays high predictability of measured ectopic fat depots, and to be part of their metabolic signatures (excluding epicardial fat). Indeed, obese individuals show increased plasma glutamate levels^49,52^.

Carbohydrate metabolism is altered in obese individuals, who show increased levels of glucose, pyruvate and lactate and most of their intermediates. The proposed causes leading to increased plasma glucose levels among obese individuals are the global adiposity, increased IR and reduced ability of adipose tissue to store glucose^49,53^. Pyruvate, metabolite important in both carbohydrate and BCAA catabolism was also reported to be increased among obese individuals. Pyruvate is converted to lactate, the major precursor of gluconeogenesis during anaerobic glycolysis which is upregulated among obese individuals. Indeed, lactate levels were found to be increased among obese individuals thus considered as an obesity-related metabolite^50,54^. In line with the obvious links between carbohydrate metabolism and obesity, we observed that the majority of metabolites related to carbohydrate metabolism had a positive association with measured ectopic fat depots. However, glucose was the only metabolite presenting significant associations with all ectopic fat depots. Moreover, our analyses highlighted glucose as a good individual classifier of measured ectopic fat depots (excluding epicardial fat), and metabolic signature component of myocardial fat.

Plasma lipidome has been extensively used to study association between circulating lipid species and obesity, due to the fact that excess body fat may lead to lipotoxicity in tissues other than adipose^55^. Accordingly, lipid abnormalities of CE, TAG, PC, LPC and SM species are often observed among obese individuals, though the behaviour of these species remains unclear and often contradictory^49,56^. For instance, in a study with weight discordant monozygotic twins, authors observed an association between obesity and increased levels of SM and LPC species and decreased levels of ether-phospholipids^57^. However, others observed a significant association between central obesity, lower LPCs but higher PC and SM levels in young adults^58^. Here, we observed that all analysed lipid classes including CE, TAG, PC, LPC and SM exhibit changes in obesity. Both SMs and ceramides are part of sphingolipid family, and as such recognized as structural components of cellular membranes and engaged in several biological functions, including cell-to-cell recognition and signalling^59,60^. Thus, disturbance of sphingolipid metabolism may promote cellular stress, mitochondrial dysfunction and insulin signalling. Hanamatsu *et al*.^59^ observed significantly increased serum levels of SM (C18:0 and 24:0) in obese individuals, together with a significant correlations between SM (C18:0, 20:0, 22:0 and 24:0) and IR, NAFLD and dyslipidaemia. The authors have not observed changes in ceramide levels, which may be due to the absence of severe inflammation among obese participants. In turn, Luukkonen *et al*.^61^ reported significant increases in all analysed ceramides in patients with NAFLD, whereas Turpin *et al*.^62^ observed elevated expression of C16 ceramide in visceral fat of obese individuals as well as strong correlation with BMI and IR. Consistently, ceramide synthase 6-deficient mice were protected against obesity, IR and adipose tissue inflammation. In line with these results, our analyses showed a positive correlation of SM 18:0 and diSM 18:0 with ectopic fat depots. Furthermore, diSM18:0, CER (d18:0/16:0) and SM (d18:2/16:0, d18:1/16:1) were highlighted as good individual classifiers and metabolic signature components of specific ectopic fat depots.

LPC is a highly abundant signalling molecule involved in different biological processes such as cellular proliferation, tumour cell invasion and inflammation^63^. Among the most abundant LPC species we observed a positive association between plasma levels of LPC 16:0, 18:0 and measured ectopic fat depots whereas LPC 18:1, 18:2 showed negative associations. Our classification analysis highlighted LPC 18:2 as a valuable individual classifier of ectopic fat depots, consistent with the observed depletion of this species in obese individuals and with the negative association with liver fat^64–66^. However, LPC 18:2 was not considered as a valuable metabolic signature component of ectopic fat depots.

Total TAG and cholesterol were previously shown to be increased but very heterogeneous among obese individuals. Rhee *et al*.^67^ observed that more saturated and shorter TAGs exhibit positive associations with IR, but not associations with longer TAGs. We have also observed a positive association between shorter TAGs and ectopic fat depots, but also negative associations with longer TAGs. Tonks *et al*.^68^ observed that several plasma lipids including DAGs, LPCs, CEs and TAG are associated with IR, where CE (16:1, 20:4) are in line with our observations. Furthermore, our analyses highlighted TAG50:1 and CE20:3 as a part of metabolic signatures of one and several measured ectopic fat depots, respectively.

Overall, metabolites from lipid metabolism (CE20:3, TAG50:1, PC38:3, LPC18:2, diSM18:0, CER (d18:0/16:0) and SM (d18:2/16:0, d18:1/16:1)) were shown to be of great value either as individual predictors or as metabolic signature components of specific ectopic fat depots.

## Conclusion

NAFLD is a multi-systemic disease caused by excessive fat accumulation in the liver, leading to impairment of this tissue. Unfortunately, fat accumulation is not limited only to liver, rather it is affecting extra-hepatic organs and quality of life in diagnosed individuals. Here we have used omics data together with computational approaches including machine learning, to identify several novel individual non-invasive biomarkers that accurately distinguish groups with high and low levels of myocardial, epicardial, pericardial, and liver fat depots. Most importantly, a combination of omics data highlighted novel fat depot-specific metabolic signatures. However, the cross-sectional nature of the study design limits inferences of causality.

## Materials and Methods

### Ethical approval

The study was approved by the Helsinki University Central Hospital Ethics Committee and conforms to the principles outlined in the Declaration of Helsinki. Each subject provided written informed consent.

### Study population

A total of 75 men were examined using the same study cohort as have been previously described^11^. Thirty-seven patients fulfilled the criteria for the metabolic syndrome^69^. In these participants, myocardial ischemia was excluded by means of adenosine stress MR perfusion test.

Exclusion criteria from the study included the following: other known acute or chronic disease based on medical history, physical examination, and standard laboratory tests (blood counts, creatinine, aspartate aminotransferase, alanine aminotransferase, thyroid-stimulating hormone), T2D (based on a 2-h oral glucose tolerance test), significant alcohol consumption (more than 20 grams per day), and treatment with other lipid lowering therapy than statins. As the hormonal status and use of contraceptives modify lipid metabolism in women, only male subjects were recruited. Smoking and elevated liver enzymes were allowed. Five study subjects were on regular medication for hypertension, three for dyslipidemia (statins), and one for both hypertension and dyslipidemia.

### Demographic variables and biochemical investigations

Body mass index (BMI) was calculated by dividing weight in kilograms by the square of the height in meters (kg/m^2^). Waist circumference was measured at a level midway between the lower rib lateral margin and the iliac crest in the horizontal position. Blood pressure was recorded as an average of five measurements obtained in the sitting position after a 5 min rest using a BPM-200 monitor (Quick Medical, WA, USA). The subjects were classified as present, past, or non-smokers.

Blood samples were collected after an overnight fast. Total serum cholesterol, TGs, and high-density lipoprotein cholesterol were measured by Konelab analyzer 60i with Konelab TM kits (both from Thermo Fisher Scientific, Finland). The concentration of low-density lipoprotein cholesterol was calculated by the Friedewald formula^70^. Fasting and postload glucose were assessed by the hexokinase method (Gluco-quant, Roche Diagnostics, Basel, Switzerland) using either a Hitachi 917 or Modular analyzer (both from Hitachi Ltd, Tokyo, Japan). Serum insulin concentration was determined by double-antibody radioimmunoassay (Pharmacia RIA kit, Pharmacia, Uppsala, Sweden). The insulin-resistance homeostasis model assessment (HOMA) index was calculated by using the formula: (fasting plasma glucose × fasting plasma insulin)/22.5^71^.

### Quantification of myocardial TG content

For measuring the myocardial TG content, cardiac ^1^H-MRS was performed in a 1.5 T MR imager (Magnetom Avanto; Siemens AG, Erlangen, Germany) using a standard flex-coil for signal reception. The spectroscopic volume of interest was placed within the interventricular septum using the end-systolic cardiac cine images in three planes. The localizer images and spectroscopic data acquisition were double-triggered to end-exhalation and end-systole, using Prospective Acquisition Correction navigator echoes (PACE, WIP-sequence, program version B17) to control for respiratory movement and ECG-derived R wave to control for cardiac pulsation. Spectral localization and data collection were performed with the PRESS sequence with 35 ms echo time, while repetition time (TR > 3000 ms) did not fall below the respiratory cycle length. Navigator echoes were collected from the lung-diaphragm interface and the end-systole triggering was set at about 80% of the resting heart rate of the subject. The spectra were collected with and without water suppression, using 32 and 4 acquisitions, respectively, and analyzed with jMRUI v3.0 software using the AMARES algorithm^72^ to determine water (4.7 ppm), methylene (1.3 ppm) and methyl (0.9 ppm) resonance areas. The myocardial TG content was expressed as a ratio of fat to water (%). Correction for methylene T2 relaxation was not possible due to lack of reliable data for cardiac application.

### Quantification of epicardial and pericardial fat

The 4-chamber oriented cine images were applied for measuring the epicardial and pericardial adipose tissue area as described previously^11^. All phases of the cine images were inspected and the measurements were performed in the end diastolic image using a standard radiologic workstation (Impax 5.5 software, Agfa Healthcare, Mortsel, Belgium). The areas of high intensity fat layers between the myocardium and the visceral pericardium (epicardial fat) and outside the parietal pericardium (pericardial fat) were measured.

### Quantification of hepatic and abdominal fat depots by MRI and ^1^H-MRS

For measuring hepatic TG content, ^1^H-MRS was performed in a 1.5 T MR imager (Magnetom Avanto; Siemens AG, Erlangen, Germany). Distribution of visceral and subcutaneous adipose tissue were measured as previously described^11,18^.

### Analysis of inflammation-associated proteome, lipidome and metabolome

Untargeted metabolomics profiling of plasma was performed by Metabolon Inc., as previously described^22^. Samples were prepared using the automated MicroLab STAR system from Hamilton Company. In brief, sample preparation was conducted using aqueous methanol extraction process to remove the protein fraction while allowing maximum recovery of small molecules. The resulting extract was divided into four fractions: one for analysis by UPLC/MS/MS (positive mode), one for UPLC/MS/MS (negative mode), one for GC/MS, and one for backup. Samples were placed briefly on a TurboVap (Zymark) to remove the organic solvent. Each sample was then frozen and dried under vacuum.

Samples were then prepared for the appropriate instrument (either UPLC/MS/MS or GC/MS). Inflammatory protein biomarkers were analyzed using the Proseek Multiplex Inflammation I^96×96^ array (Olink Bioscience, Uppsala, Sweden).

For lipidomics, plasma was extracted using butanol/methanol (BUME) as previously described^73^. Internal standards (either deuterated or containing heptadecanoyl (C17:0) fatty acids) were added during the extraction. Analysis of CE, TG, PC, LPC and SM were made using direct infusion on a QTRAP 5500 mass spectrometer (Sciex, Concord, Canada) equipped with a robotic nanoflow ion source, the TriVersa NanoMate (Advion BioSciences, Ithaca, NJ). Detection of cholesteryl esters was made using precursor ion scanning in positive mode using *m/z* 369 (protonated cholestadiene) as fragment ion^74^. Detection of TG was made using neutral loss scanning in positive mode^75^. Phospholipids were analysed in both positive and negative ion mode using (multiple) precursor ion scanning^76,77^. After alkaline hydrolysis in 0.1 M potassium hydroxide in methanol, sphingomyelin was analysed in positive mode using *m/z* 184 (phosphocholine) as fragment ion. Quantification of lipids was made using a one point calibration against the added, lipid class specific, internal standard. Data was evaluated using LipidView software.

### Statistical analyses

All statistical analyses were conducted in R 3.2.1 for Mac (R Development Core Team, 2015). Categorical variables of myocardial, epicardial, pericardial and liver fat were defined using 33% of high and low values. All continuous variables with more than 5% of missing values were excluded from the analysis. Continuous variables were analysed for the normality with Shapiro-Wilk normality test and Leven’s test was used to assess the groups equality of variances (data not shown). Between group differences (high = 25; low = 25) were examined using Mann-Whitney U test (i.e. Wilcoxon Rank-Sum test). In order to assess the relationship between continuous variables Spearman correlation factor was calculated. Classification analysis/variable selection was conducted using random forest (RF) machine learning algorithm. RF modelling is appealing technique for high-dimensional data, shown to identify important covariates with respect to the variable of interest. Even though RF can be run with default settings it is common by the users to tune certain parameters in order to obtain more stable and reproducible model. For the same reason the number of growing trees was set to 100,000 while all other parameters were left unchanged^78^. RF algorithm is constructing each tree from the new training set, drawn with replacement from the original data. About one-third of the training set are left out and used as a test set (i.e. out of bag, OOB) upon which unbiased estimate of classification error rate is calculated. The study conducted by Breiman^79^ presented empirical evidence that OOB estimate of classification error is as accurate as using an independent test set thus, removing the need for the same. A detailed description of the RF algorithm and its optimization can be found elsewhere^80^. Area under the Receiver Operating Characteristic (ROC) curves (AUC) were used to estimate the diagnosis ability of individual biomarkers obtained from RF as a classifier in a clinical application. An optimal criterion was defined for each potential biomarker by maximizing both true positive (i.e. sensitivity, SN) and true negative (i.e. specificity, SP) rates, where higher AUC indicate better classifications^78,81^. All P-values are reported after FDR correction (Benjamin-Hochberg) with significance level of 0.05.

## Electronic supplementary material

Table 1

Table 2

Table 3

Table 4

## Data Availability

The data that supports the findings of this study are available from the corresponding author upon request.
